# Healthcare decision-making in end stage renal disease-patient preferences and clinical correlates

**DOI:** 10.1186/s12882-015-0180-8

**Published:** 2015-11-14

**Authors:** Anuradha Jayanti, Markus Neuvonen, Alison Wearden, Julie Morris, Philip Foden, Paul Brenchley, Sandip Mitra, on behalf of the BASIC-HHD study group

**Affiliations:** Department of Nephrology, Central Manchester Hospitals NHS Trust, Manchester Royal Infirmary, Oxford Road, Manchester, M13 9WL UK; Department of Political and Economic Studies, University of Helsinki, Helsinki, Finland; Department of Psychology, University of Manchester, Manchester, UK; Department of Biostatistics, University of Manchester, Manchester, UK

**Keywords:** Haemodialysis, Autonomy, Decision-making, Cognition

## Abstract

**Background:**

Medical decision-making is critical to patient survival and well-being. Patients with end stage renal disease (ESRD) are faced with incrementally complex decision-making throughout their treatment journey. The extent to which patients seek involvement in the decision-making process and factors which influence these in ESRD need to be understood.

**Methods:**

535 ESRD patients were enrolled into the cross-sectional study arm and 30 patients who started dialysis were prospectively evaluated. Patients were enrolled into 3 groups- ‘predialysis’ (group A), ‘in-centre’ haemodialysis (HD) (group B) and self-care HD (93 % at home-group C) from across five tertiary UK renal centres. The Autonomy Preference Index (API) has been employed to study patient preferences for information-seeking (IS) and decision-making (DM). Demographic, psychosocial and neuropsychometric assessments are considered for analyses.

**Results:**

458 complete responses were available. API items have high internal consistency in the study population (Cronbach’s alpha > 0.70). Overall and across individual study groups, the scores for information-seeking and decision-making are significantly different indicating that although patients had a strong preference to be well informed, they were more neutral in their preference to participate in DM (p < 0.05). In the age, education and study group adjusted multiple linear regression analysis, lower age, female gender, marital status; higher API IS scores and white ethnicity background were significant predictors of preference for decision-making. DM scores were subdivided into tertiles to identify variables associated with high (DM > 70: and low DM (≤30) scores. This shows association of higher DM scores with lower age, lower comorbidity index score, higher executive brain function, belonging in the self-caring cohort and being unemployed. In the prospectively studied cohort of predialysis patients, there was no change in decision-making preference scores after commencement of dialysis.

**Conclusion:**

ESRD patients prefer to receive information, but this does not always imply active involvement in decision-making. By understanding modifiable and non-modifiable factors which affect patient preferences for involvement in healthcare decision-making, health professionals may acknowledge the need to accommodate individual patient preferences to the extent determined by the individual patient factors.

**Electronic supplementary material:**

The online version of this article (doi:10.1186/s12882-015-0180-8) contains supplementary material, which is available to authorized users.

## Background

*‘Nothing is more difficult, and therefore more precious, than to be able to decide’*

*Napoleon Bonaparte*

Medical decision-making is critical to patient survival and well-being[1]. Over the last two decades, the convergence of influential ideas from the fields of bioethics, psychology, sociology and medicine has contributed to our understanding of the beneficial role of engaging patients in the medical decision-making process. The several potential benefits of involving patients in medical decision-making (DM) include reduced anxiety and depression, greater self-efficacy, improved concordance, and higher satisfaction with their physician [2–6]. Patient’s expectations about exercising choice in medical decision-making have also been influenced by socio-cultural factors. These stem from increasing consumerist attitudes and litigious practices in the society, leading to the belief amongst healthcare professionals, that patients are best placed to evaluate the risks and benefits of alternative treatments [7], [8]. Of the models of healthcare decision-making that exist, an extreme and impractical version of the ‘patient engagement model’ of healthcare in practice would result in the providers supplying accurate information to patients without sharing their own views or experiences and then expecting patients to make tough medical decisions on their own. Research has demonstrated that patients’ desire for information is typically underestimated by physicians[6]. What is less apparent is the extent to which they seek involvement in the actual decision-making process.

Healthcare decision-making is a highly complex process, the outcome of which is the interplay of several interrelated factors[7, 9, 10] and not limited only to uncertainty in scientific evidence. As decision-making is affected by several factors, it is prone to error[1]. It is not surprising therefore, as to why some patient decisions may be at odds with the healthcare provider recommendations, making even shared decisions difficult to implement in clinical practice.

In several clinical conditions, evidence shows that not all patients want to make their own decisions[6] and some would actively delegate the task to their healthcare professionals. This concept has not been well understood in chronic kidney disease (CKD). Patients with CKD are faced with incrementally complex decision-making throughout their treatment journey. Particularly in later stages, patients exercise choice and make decisions which impact on how they live from day to day. Some of these include decisions around dietary intake, medications, frequency of clinic visits, treatment options when they reach end stage renal disease (ESRD) and even the choice of not considering renal replacement therapy. Dialysis, a life-sustaining therapy, invites multiple levels of patient engagement with and without healthcare providers, making it an intellectually and emotionally demanding process. Accommodating individual patient preferences for participation and true shared decision-making as the ‘ideal’ may be in potential conflict in some instances.

‘Autonomy’ in decision-making is one of several factors which may influence healthcare decisions throughout the ESRD journey. We chose to study this construct to understand its basis in undertaking self-care in the ESRD context. Decisions are taken based on the information patients acquire from healthcare providers and other sources. Also, the impact of ‘real’ vs ‘imaginary’ knowledge may influence patient attitudes to decision-making. Patients with ESRD are expected to assimilate a lot of new information in a particularly vulnerable phase of their illness, sometimes with limitations in cognitive and computational skills [11] and in relatively short time frames, leading to critical, life-changing decisions. Multiple inter-related skills are required to function optimally and produce the best outcomes for the individual circumstance. These include the ability to access and comprehend information, recall the same, weigh alternatives, infer and communicate decisions effectively and engage in a life-long process of learning [12, 13]. All of these activities are a product of complex processing of information in the brain of individuals. Executive brain function is a higher order cognitive ability that is a product of working memory, reasoning, task flexibility and visuo-motor speed. It is well known that chronic kidney disease is associated with considerable executive and episodic memory cognitive deficit, which is also progressively on the decline, after commencement of haemodialysis [11]. The reported prevalence of cognitive deficit in dialysis patients is of the order of 17-50 % [14]. Closely related to systematic, careful cognitive processing is the role of the patient’s ‘affect’ on decision-making. Understanding ESRD healthcare decision-making from a psychological perspective is paramount due to the high prevalence of depression or anxiety amongst these patients (up to 70 %) [15, 16].

### Study objectives

Although ‘autonomy’ and ‘decision-making’ are not synonymous with each other, in contemporary medical literature, the two have been used interchangeably[24]. In the present study, ‘information-seeking’ and ‘decision-making’ preferences are evaluated in a large group of ESRD patients.

We sought to

1. Describe the properties of Autonomy Preference Index (API) instrument in ESRD population.
2. Examine clinical, psychological and neurocognitive correlates of ‘autonomous decision-makers’ vs ‘delegators’ in ESRD.
3. Study the impact of commencement of dialysis on decision-making, in a subset of predialysis patients.

## Methods

The API study data are derived from data ascertained for the BASIC-HHD study[18]. The BASIC-HHD study is a comprehensive and systematic study of barriers and enablers of the uptake and maintenance of home HD therapy. The study involves five UK centres, with variable prevalence rates of home HD. The centres reported similar structure of pre-dialysis education programmes with access to nurse specialists for information and dedicated ‘low-clearance’ clinics. An integrated mixed methodology (convergent, parallel design) has been adopted for the BASIC-HHD study in a combined cross-sectional and prospective study design. The methodological details and scope of data collected in the BASIC-HHD appear in a published protocol[18].

### Study registration

This study has been reviewed and approved by the Greater Manchester West Health Research Authority National Research Ethics Service (NRES) Reference number: 12/NW/0170. The study is on the NIHR portfolio (ID 12346). Written, informed consent from participants was obtained for the study

### Participants

Data presented here are derived from the cross-sectional and prospective segments of the BASIC-HHD study. 535 patients were enrolled in three groups. Predialysis patients for the CKD-5 group (group A), prevalent ‘in-centre’ HD patients (group B) were approached if they fulfilled eligibility criteria and were willing to undertake neuropsychometric assessments and complete study specific questionnaires. All self-care haemodialysis patients (93 % at home) from each participating centre were also approached (group C). Predialysis patients were approached consecutively from the predialysis clinics and hospital haemodialysis patients were approached in consecutive order across all shifts until the centre target for recruitment was reached. Most participants approached were willing to engage with the study and reasons for declining participation included a lack of interest in research participation, and ‘research’ fatigue.

### Procedure

Psychological measures employed in this study were a part of compilation of questionnaires. Blood sampling and neuropsychometric assessments were carried out ahead of the dialysis sessions. HD patients returned the questionnaires on the same day or within a couple of dialysis sessions ‘in-centre’. Home HD patients returned it by post, as did the pre-dialysis patients. Visually impaired patients could respond to questions posed to them by the research team member.

### Measures

The Autonomy Preference Index was used to study patient preferences for information-seeking and decision-making. This tool was developed and validated originally in a group of general medical patients [17]. This tool consists of two subscales: an eight-item information-seeking subscale and a six-item decision-making subscale. The format of the responses is on a 5-point Likert scale. Scores for both domains are linearized to range from 0–100 (percentage scores), with higher scores indicating stronger preferences for participation. In addition, in the original API there are eight items corresponding to three clinical vignettes representing increasing disease severity to assess if symptom severity plays a role in patient autonomy preferences. The API has been validated and utilized in numerous other patient populations. The tool was employed; unmodified, as the questions and clinical scenarios are both relevant and not unfamiliar to the ESRD population.

Additionally, all study participants completed a compilation of questionnaires[18]. In order to examine the potential impact of patient’s affect and cognitive ability on their engagement with decision-making, additional instruments analysed in the present study are the Beck Depression Inventory II[19] and the State and Trait Anxiety Inventory[20]. Participants underwent cognitive assessment using the modified mini-mental state examination (3MS)[21], and trail making tests A and B (TMTA/TMTB) scores[22]. The scores from these instruments were considered in ordered categories for analyses: BDI (0–10, 11–15, 16–20, 21–25, 26–30, 31+), STAI (20–29, 30–39, 40–49, 50+) and 3MS (94–100, 86–93, 81–85, 76–80, ≤75).

### Missing data

**Fig. 1:**
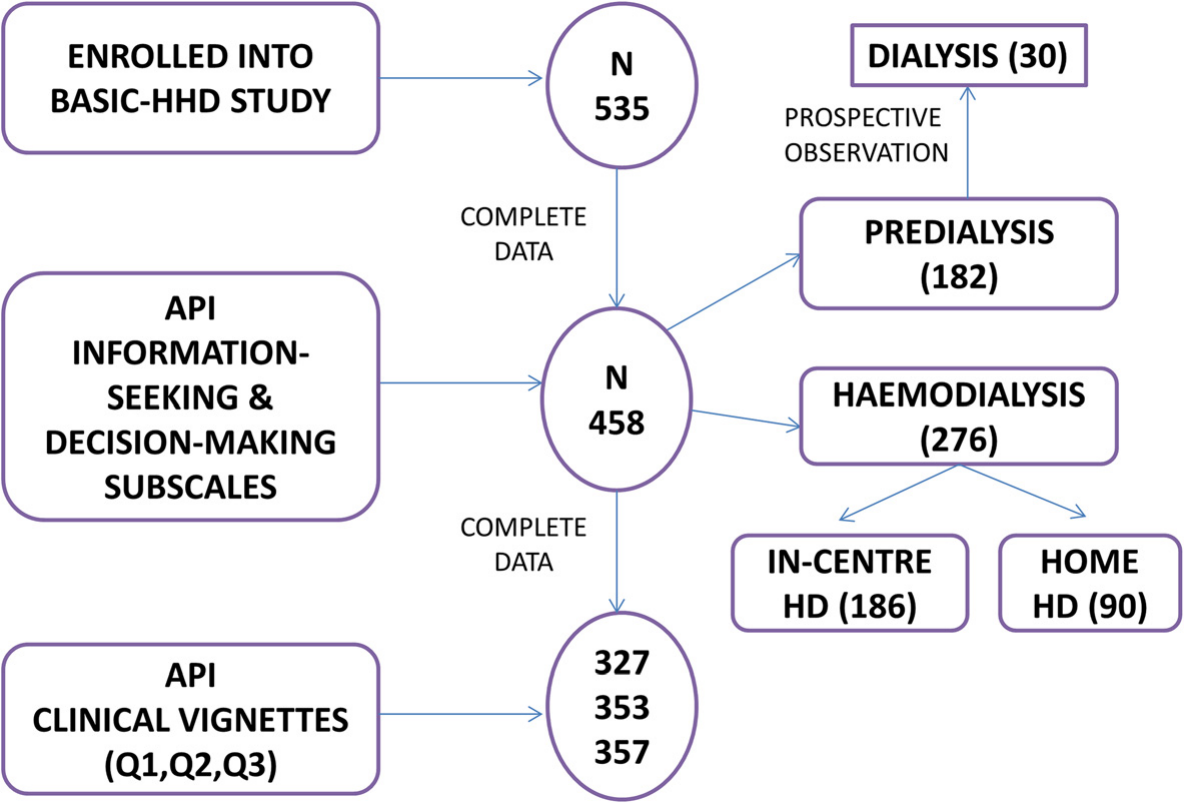
Diagram depicting API data available for analysis (N)

Overall the study had excellent data completion across all instruments used in the study (>82 %). The API subscales were complete in 85.6 % of the cases (Fig. 1). The only statistically significant difference between those who were missing both the API decision-making and API information-seeking scores (n = 77) and those who were not missing both is in ethnicity. Non-white patients were more likely not to complete both API scores than white patients. Ethnicity was associated with decision making in the final multivariable analysis for the decision making variable. Therefore, there is a chance that the point estimate may change slightly, depending on whether the non-white patients who responded had different scores to those who did not respond. However, with the relatively small amount of missing data and only 15 non-white patients not having either score, any change would be small. There was no relationship in the single variable analysis between ethnicity and information seeking so unless the missing non-white patients differed greatly to the non-white patients who responded, it is likely the lack of association would remain (see Additional file 1: supplementary information).

### Statistical analyses

All analyses were carried out using SPSS 20. Patient characteristics between groups were compared using ANOVAs, chi-square tests and Kruskal-Wallis tests using conventional two-sided 5 % significance level. Appropriate adjustments – Scheffé adjustments for pairwise differences in ANOVA, Bonferroni adjustments in z-tests of category proportions and Mann–Whitney U tests – were made to account for multiple testing when carrying out pairwise comparisons. Cronbach’s alpha was used to assess the internal consistency of the two API subscales in the ESRD group. A confounder-adjusted analysis has been carried out for all variables considered in the study in accordance with the definition of a potential confounder[23]. Variables from this analysis were also used to inform the choice of variables in the multivariable regression model. The multivariable linear regression with a backwards step-wise selection method was used to identify the variables that are associated with the API decision-making preference of patients. The same selection method was used for API information seeking. Variables with p-value of less than 0.15 in the single variable analysis were considered for selection in the multivariable analysis. In the multivariable analysis, three variables were considered clinically important: age, education and group. The other variables were removed from the model until only those with a p-value less than 0.05 remained. A linear mixed effects model with centre as the random effect has been used to account for a possible centre effect .The ICC (intraclass correlation coefficient) is the measure of the ratio of the between cluster variance to the total variance (between-cluster + within-cluster). ICC close to 1 indicates the people in the cluster are very similar, whereas ICC close to 0 indicates the between-cluster variability is small compared to the within-cluster variability. Three patient subgroups, based on the API decision-making scores were also constructed, to understand factors associated with these scores in the highest and lowest tertiles. The patient subgroup characteristics were examined using Mann–Whitney U tests, Fisher’s exact tests and linear-by-linear chi-square tests. Paired t-tests were used to examine change over time for the prospective data from 30 Group ‘A’ (CKD-5) patients.

## Results

### Demographic and clinical characteristics of the ESRD population

A total of 458 responses were available. 39.7 % of the responses came from predialysis patients. Overall, patients receiving home HD were younger, more educated and in employment. They had the support of care-givers at home, predominantly, spouses. There were significantly greater numbers of patients with diabetes and greater comorbidity burden in the ‘predialysis’ and ‘in-centre’ dialysis groups. There was no significant difference between groups with respect to previously diagnosed affective disorders or in their screening for anxiety and depression using validated inventories. These group comparisons are important to adjust later analyses for potential confounders. The differences between the groups are illustrated in Table 1. Due to clinical importance and due to the fact there were differences between the groups, it was automatically included as a variable in both the API-DM and API-IS analyses. The group variable being included should therefore account for the differences in characteristics of the group.

**Table 1:** Demographic, clinical and psychosocial characteristics of the ESRD study population

Variable		Total (*n* = 458)	Predialysis(A) (*n* = 182)	In-centre HD(B) (*n* = 186)	Home HD(C) (*n* = 90)	*p*-value
Age Mean (std. dev.)			59.84 (13.28)	56.84 (14.78)	51.81 (11.67)	*p* < 0.001^a^ A > C *p* < 0.001^b^ B > C *p* = 0.017^b^
Gender	Male	296 (64.6 %)	110 (60.4 %)	119 (64.0 %)	67 (74.4 %)	*p* = 0.073^c^
Education	Post high school education	111 (25.1 %)	43 (24.2 %)	31 (17.5 %)	37 (42.5 %)	*p* < 0.001^c^ A < C *p* < 0.05^d^ B < C *p* < 0.05^d^
Employment	Retired	213 (46.8 %)	94 (51.6 %)	87 (47.3 %)	32 (36.0 %)	*p* < 0.001^c^ Retired
	Unemployed	115 (25.3 %)	31 (17.0 %)	64 (34.8 %)	20 (22.5 %)	A > C *p* < 0.05^d^ Unemployed
	Self-employed	35 (7.7 %)	17 (9.3 %)	10 (5.4 %)	8 (9.0 %)	A < B *p* < 0.05^d^
	Salaried	92 (20.2 %)	40 (22.0 %)	23 (12.5 %)	29 (32.6 %)	Salaried A > B *p* < 0.05^d^ C > B *p* < 0.05^d^
Ethnicity	Non-white	46 (10.1 %)	14 (7.7 %)	20 (10.8 %)	12 (13.3 %)	*p* = 0.32^c^
BMI (kg/m^b^) Median (interquartile range)			28.38 (24.27, 32.45)	26.50 (23.08, 31.51)	26.53 (23.63, 30.83)	*p* = 0.031^e^ A > B *p* = 0.040^f^ A > C *p* = 0.21^f^ C > B *p* = 1.00^f^
Smoking status	Never smoked	257 (56.7 %)	98 (54.1 %)	103 (56.6 %)	56 (62.2 %)	*p* = 0.57^c^
	Ex-smoker	133 (29.4 %)	54 (29.8 %)	53 (29.1 %)	26 (28.9 %)	
	Current	63 (13.9 %)	29 (16.0 %)	26 (14.3 %)	8 (8.9 %)	
Caregiver	Spouse/partner	250 (56.2 %)	111 (62.0 %)	83 (46.6 %)	56 (63.6 %)	*p* = 0.004^c^ Spouse/part
	Child carer	24 (5.4 %)	12 (6.7 %)	6 (3.4 %)	6 (6.8 %)	A > B *p* < 0.05^d^ C > B *p* < 0.05^d^
	Parent carer	34 (7.6 %)	10 (5.6 %)	16 (9.0 %)	8 (9.1 %)	F, R, S or C
	Friend, relative, sibling or carer	19 (4.3 %)	3 (1.7 %)	13 (7.3 %)	3 (3.4 %)	B > A *p* < 0.05^d^
	Alone	118 (26.5 %)	43 (24.0 %)	60 (33.7 %)	15 (17.0 %)	Alone B > C *p* < 0.05^d^
Marital status	Married	253 (55.2 %)	106 (58.2 %)	89 (47.8 %)	58 (64.4 %)	*p* = 0.020^c^ Married B < C p < 0.05^d^
	Partner	27 (5.9 %)	11 (6.0 %)	10 (5.4 %)	6 (6.7 %)	
	Single	103 (22.5 %)	38 (20.9 %)	54 (29.0 %)	11 (12.2 %)	Single B > C p < 0.05^d^
	Divorced or separated	40 (8.7 %)	11 (6.0 %)	17 (9.1 %)	12 (13.3 %)	
	Widowed	35 (7.6 %)	16 (8.8 %)	16 (8.6 %)	3 (3.3 %)	
Psych usage	Never offered	180 (40.8 %)	70 (38.9 %)	84 (48.8 %)	26 (29.2 %)	*P* < 0.001^c^ Never offered B > C *p* < 0.05^d^ Never used A > B *p* < 0.05^d^ Used and found useful C > A *p* < 0.05^d^ Used but not useful C > B *p* < 0.05^d^
	Never used	209 (47.4 %)	98 (54.4 %)	69 (40.1 %)	42 (47.2 %)	
	Used and found useful	36 (8.2 %)	6 (3.3 %)	16 (9.3 %)	14 (15.7 %)	
	Used but not useful	16 (3.6 %)	6 (3.3 %)	3 (1.7 %)	7 (7.9 %)	
Primary cause of ESRD	Hypertensive Nephrosclerosis	55 (12.0 %)	34 (18.7 %)	12 (6.5 %)	9 (10.0 %)	*p* < 0.001^c^ Hyp Neph A > B *p* < 0.05^d^
	Diabetic Nephropathy	96 (21.0 %)	45 (24.7 %)	42 (22.6 %)	9 (10.0 %)	Diab Neph A > C *p* < 0.05^d^ B > C *p* < 0.05^d^
	Glomerulonephritis	65 (14.2 %)	19 (10.4 %)	30 (16.1 %)	16 (17.8 %)	Polycystic KD C > A *p* < 0.05^d^ C > B *p* < 0.05^d^
	Polycystic Kidney Disease	55 (12.0 %)	18 (9.9 %)	18 (9.7 %)	19 (21.1 %)	
	Renovascular Disease	12 (2.6 %)	4 (2.2 %)	8 (4.3 %)	0 (0 %)	
	Chronic Pyelonephritis/ Reflux Nephropathy	29 (6.3 %)	8 (4.4 %)	15 (8.1 %)	6 (6.7 %)	
	Others	83 (18.1 %)	36 (19.8 %)	32 (17.2 %)	15 (16.7 %)	
	Unknown	63 (13.8 %)	18 (9.9 %)	29 (15.6 %)	16 (17.8 %)	
	Yes	121/276 (43.8 %)	~	83 (44.6 %)	38 (42.2 %)	
Hypertension	Yes	348 (76.0 %)	152 (83.5 %)	123 (66.1 %)	73 (81.1 %)	*p* < 0.001^c^ A > B *p* < 0.05^d^ C > B *p* < 0.05^d^
Diabetes	Yes	123 (27.0 %)	56 (30.8 %)	56 (30.4 %)	11 (12.4 %)	*p* = 0.002^c^ A > C *p* < 0.05^d^ B > C *p* < 0.05^d^
H/O Anxiety	Yes	14 (3.1 %)	5 (2.7 %)	5 (2.7 %)	4 (4.4 %)	*p* = 0.69^c^
H/O Depression	Yes	48 (10.5 %)	17 (9.3 %)	18 (9.7 %)	13 (14.4 %)	*p* = 0.39^c^
CCI Median (inter-quartile range)			5.00 (3.75, 6.00)	4.00 (3.00, 6.00)	3.50 (2.00, 5.00)	*p* < 0.001^e^ A > B *p* = 0.60^f^ A > C *p* < 0.001^f^ B > C *p* = 0.004^f^
BDI	0-10	239 (52.2 %)	102 (56.0 %)	91 (48.9 %)	46 (51.1 %)	*p* = 0.783
	11-15	73 (15.9 %)	27 (14.8 %)	31 (16.7 %)	15 (16.7 %)	
	16-20	45 (9.8 %)	18 (9.9 %)	20 (10.8 %)	7 (7.8 %)	
	21-25	44 (9.6 %)	17 (9.3 %)	20 (10.8 %)	7 (7.8 %)	
	26-30	25 (5.5 %)	6 (3.3 %)	13 (7.0 %)	6 (6.7 %)	
	≥31	32 (7.0 %)	12 (6.6 %)	11 (5.9 %)	9 (10.0 %)	
STAI State	20-29	148 (33.4 %)	54 (30.0 %)	61 (34.5 %)	33 (38.4 %)	*p* = 0.863
	30-39	131 (29.6 %)	57 (31.7 %)	53 (29.9 %)	21 (24.4 %)	
	40-49	100 (22.6 %)	42 (23.3 %)	38 (21.5 %)	20 (23.3 %)	
	≥50	64 (14.4 %)	27 (15.0 %)	25 (14.1 %)	12 (14.0 %)	
STAI Trait	20-29	121 (27.6 %)	47 (26.3 %)	49 (28.2 %)	25 (29.1 %)	*p* = 0.433
	30-39	124 (28.2 %)	46 (25.7 %)	54 (31.0 %)	24 (27.9 %)	
	40-49	111 (25.3 %)	55 (30.7 %)	39 (22.4 %)	17 (19.8 %)	
	≥50	83 (18.9 %)	31 (17.3 %)	32 (18.4 %)	20 (23.3 %)	
TMT A Median (inter-quartile Range)			46.65 (32.25, 60.00)	47.00 (36.00, 69.00)	35.50 (30.00, 47.53)	*p* < 0.001^e^ B > A *p* = 0.39^f^ A > C *p* = 0.001^f^ B > C *p* < 0.001^f^
TMT B Median (inter-quartile range)			90.00 (68.50, 120.00)	113.00 (73.00, 145.00)	74.00 (61.00, 94.00)	*p* < 0.0015 B > A *p* = 0.0236 A > C *p* = 0.0076 B > C *p* < 0.0016
3MS	≤75	11 (2.6 %)	2 (1.2 %)	8 (4.6 %)	1 (1.3 %)	*p* = 0.0517
	76-80	14 (3.3 %)	6 (3.5 %)	6 (3.5 %)	2 (2.6 %)	
	81-85	35 (8.3 %)	9 (5.3 %)	21 (12.1 %)	5 (6.5 %)	
	86-93	157 (37.3 %)	64 (37.4 %)	69 (39.9 %)	24 (31.2 %)	
	94-100	204 (48.5 %)	90 (52.6 %)	69 (39.9 %)	45 (58.4 %)	

^a^ANOVA p-value for overall between groups mean differences

^b^Scheffe adjusted p-values for comparison of pair-wise group means

^c^Pearson Chi-Square p-value

^d^z-test comparing category proportions between groups, p-value with Bonferroni adjustment for multiple testing

^e^Kruskal-Wallis test p-value

^f^Mann-Whitney *U* test p-value with adjustment for multiple testing

### API in ESRD population

We measured the internal consistency of the items in the API, in our study population using Cronbach’s alpha. This was acceptable for both information-seeking (Cronbach’s alpha = 0.774) and decision-making (Cronbach’s Alpha = 0.714) subscales of the API. The mean and standard deviations for all the items in both subscales are presented in the Additional file 2: supplementary material.

### Descriptive data analysis of the two subscales and clinical vignettes

**Fig. 2:**
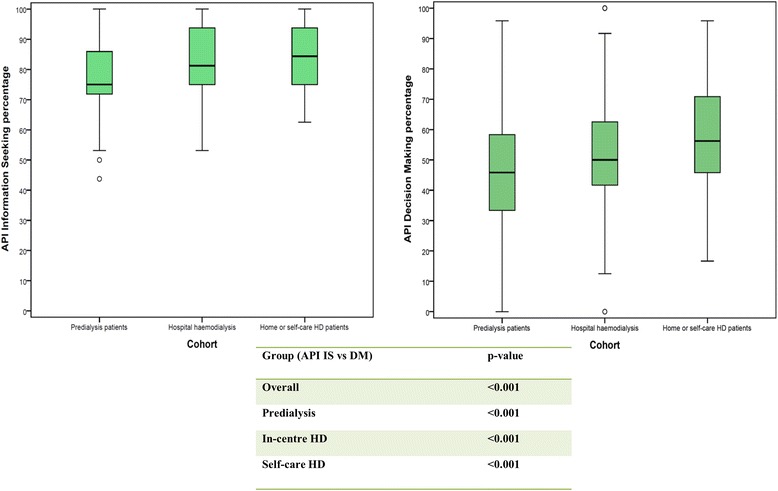
Box Plots showing the median scores on the API for Information-seeking and Decision-making subscales in all three study groups

**Fig. 3:**
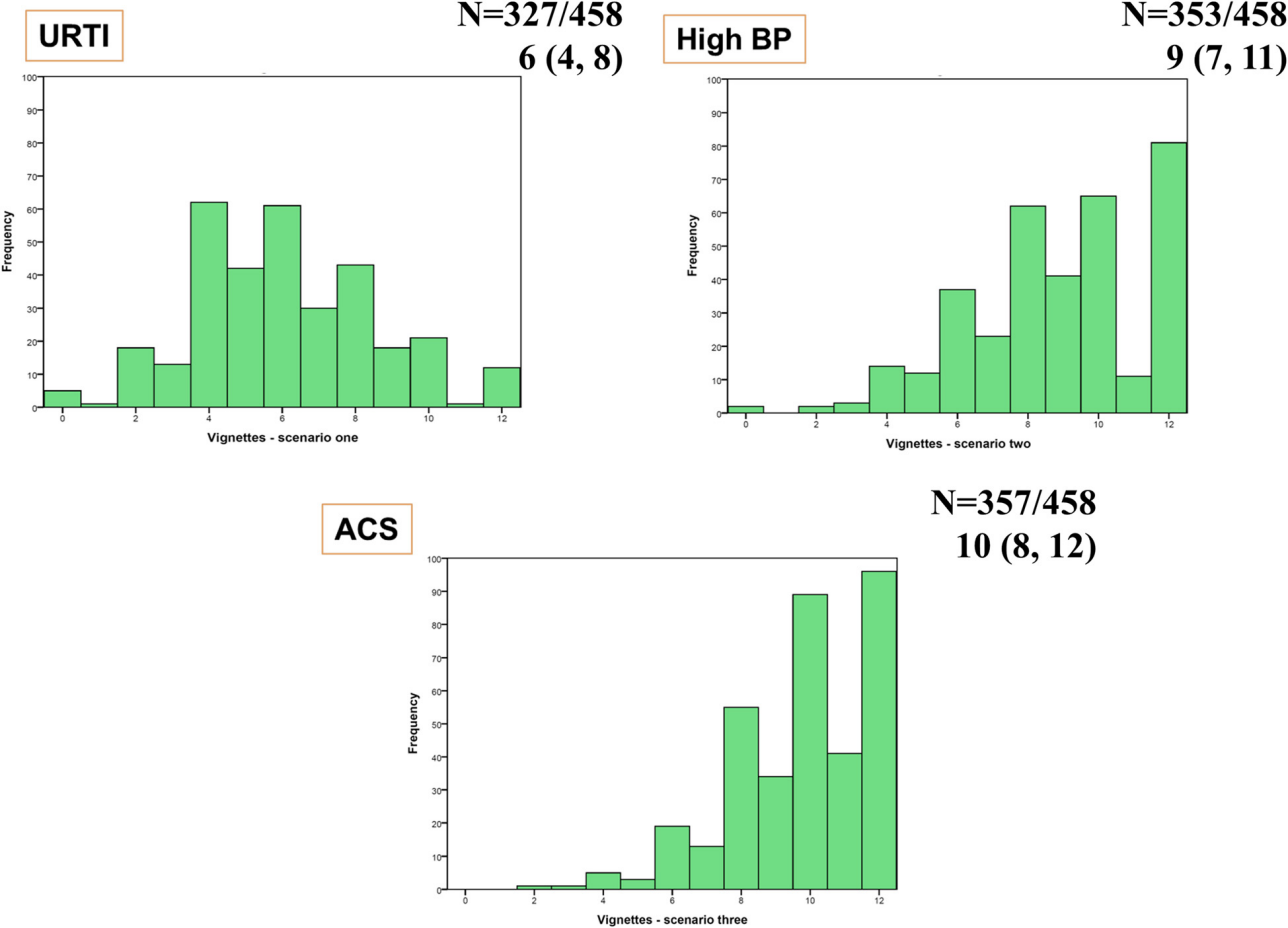
Responses to the three clinical vignettes from the API tool by the ESRD group. Actual scores are presented on the x-axis and frequency distribution of the scores is presented along the y-axis. The responses patients could choose from are provided in the API tool in the Additional file 3: supplementary material. Vignette 1: Patient preference for management of a simple upper respiratory tract infection (URTI). Median score 6 (Interquartile range 4, 8). Vignette 2: Patient preference for management of high blood pressure (BP). Median score 9 (Interquartile range 7, 11). Vignette 3: Patient preference for management of a heart attack or acute coronary syndrome (ACS). Median Score 10 (Interquartile range 8, 12)

The median score for the API information-seeking scale and the API decision-making scale in all three study cohorts is depicted in box plots (Fig. 2). For the API clinical vignettes, worsening symptom severity was associated with a change in treatment decision-making preference scores, and most patients in the collective ESRD group wanted shared decision-making with their healthcare provider if symptoms hypothetically worsened (Fig. 3).

### Demographic, clinical and psychosocial factors predicting decision-making in ESRD

In the single variable analysis of the information-seeking subscale scores, the predictors at the 15 % significance level of high IS scores were (linear regression with just the variable of interest in the model): age, education, study group, gender, marital status, heart failure, BDI score, 3MS, IMD score (index of multiple deprivation), first choice of dialysis modality and perceived ability to consider self-cannulation for haemodialysis.

In the single variable analysis of the decision-making subscale (Table 2), variables significant at the 5 % level (a linear regression with just the variable of interest in the model) and were considered for the multivariable analysis include: age, study group, employment, marital status, psychology service use, diabetes, heart failure, ischaemic heart disease history, CCI, TMT A and API information-seeking subscale score. Additionally, variables up to 15 % significance were also included in the multivariable model. These include gender, ethnicity, informal caregiver availability and patient attendance of a treatment options education session. The confounder adjusted analysis has highlighted a number of significant variables in common with the unadjusted single variable analysis. The multiple linear regression selection process for the decision-making subscale (Table 3), which had age, education and study group adjusted for, selected lower age, female gender, marital status, API information-seeking scores and white ethnicity background at 5 % significance level, in favour of greater autonomy in decision-making. Education was not significant in the multivariable analysis. The multiple linear regression selection process for the information-seeking scores (Table 3), which had age, education and study group adjusted for, selected lower age, post high school education, marital status and per category increase in BDI score at the 5 % significance level as significantly associated with information-seeking. In the model where ‘centre-effect’ was evaluated, sensitivity analysis that suggests very little change for API-DM and for API-IS education and age are slightly less significant than in the model without the centre-effect included. The likelihood ratio test in both cases failed to reject the null hypothesis of there being no difference between the mixed effects model and the ordinary linear model.

**Table 2:** Single variable analysis and confounder adjusted analysis (Decision-making)

Single Variable Analysis (API-DM)				Confounder Adjusted Analysis (API-DM)		
Variable of interest		EMM* (95 % CI)	*p*-value	Confounders	Regression Coefficient (95 % CI)	*p*-value
Age (per year)**		71.60-0.37 (−0.48, −0.26)	<0.001	Ethnicity, Caregiver, Marital status, Education session, Psych service use, API-IS, group	−0.33 (−0.48, −0.19)	<0.001
Education	High school	49.88 (47.96, 51.80)	0.29	Age, ethnicity, psych service use, diabetes, education session	−2.31(−6.12, 1.50)	0.23
	Post high school	51.91 (48.64, 55.19)				
Gender	Male	49.49 (47.44, 51.53)	0.092	CCI	−2.03 (−5.47, 1.41)	0.25
	Female	52.45 (49.67, 55.24)				
Employment	Retired	46.05 (43.69, 48.42)	<0.001	Ethnicity, caregiver, marital status, psych service use, CCI, education session, TMT A	−2.61 (−7.65, 2.42) 7.97 (2.63, 13.30) 0.04 (−7.47, 7.55)	0.001
	Unemployed	57.13 (53.94, 60.32)				
	Self employed	50.00 (44.25, 55.75)				
	Salaried	56.63 (49.08, 56.18)				
	Employed	51.90 (48.79, 55.02)				
Ethnicity	White	50.90 (49.16, 52.64)	0.13	Age, employment	8.22 (2.86, 13.59)	0.003
	Non-white	46.65 (41.50, 51.80)				
BMI	<25	49.89 (47.08, 52.71)	0.74	Age, gender	−2.58 (−6.42, 1.26) −0.06 (−4.05, 3.93)	0.33
	25-29.99	50.12 (47.14, 53.09)				
	≥30	51.37 (48.52, 54.23)				
Smoking Status	Never smoked	51.19 (48.97, 53.40)	0.65	Age, gender, employment	1.98 (−2.81, 6.76) 2.50 (−2.78, 7.79)	0.64
	Ex-smoker	49.39 (46.30, 52.48)				
	Current	50.33 (45.90, 54.77)				
Caregiver	Spouse or partner	50.31 (48.07, 52.54)	0.12	Employment, psych service use, CCI, group	3.56 (−0.44, 7.55) −2.08 (−9.89, 5.72) 1.66 (−5.55, 8.88) 3.28 (−5.15, 11.71)	0.33
	Child	46.56 (39.26, 53.86)				
	Parent	57.35 (51.35, 63.36)				
	Friend, Relative Sibling, Carer	53.47 (45.22, 61.72)				
	Alone	49.05 (45.82, 52.27)				
Marital Status	Married or partner	51.22 (49.12, 53.31)	0.006	Age, employment, psych service use, TMT A, group	7.88 (1.51, 14.25) 3.95 (−3.40, 11.31) 4.29 (−3.95, 12.53)	0.041
	Single	51.86 (48.44, 55.28)				
	Divorced or separated	51.28 (45.73, 56.84)				
	Widowed	40.36 (34.49, 46.22)				
Psych service use	Not used	49.75 (47.96, 51.54)	0.088	Age, employment, caregiver, marital status, diabetes, TMT A, group	0.56 (−5.13, 6.24)	0.85
	Used	54.25 (49.40, 59.09)				
Diabetes	No	51.63 (49.70, 53.55)	0.021	Age, employment, psych service use	2.75 (−0.95, 6.46)	0.14
	Yes	47.21 (43.99, 50.43)				
Heart Failure	No	50.91 (49.23, 52.60)	0.042	Age	5.61 (−1.76, 12.99)	0.14
	Yes	42.99 (35.54, 50.44)				
IHD	No	51.51 (49.64, 53.39)	0.032	Age, gender, employment, diabetes, TMT A	−0.50 (−4.68, 3.68)	0.81
	Yes	47.24 (43.81, 50.66)				
Stroke	No	50.79 (49.07, 52.51)	0.28	Age, IHD, education session	−0.05 (−6.13, 6.04)	0.99
	Yes	47.38 (41.46, 53.31)				
Solid Organ Malignancy	No	50.26 (48.51, 52.01)	0.36	Age, employment, caregiver, psych service use, diabetes, group	−2.71 (−8.09, 2.67)	0.32
	Yes	52.78 (47.72, 57.84)				
Charlson Comorbidity Index (per unit)**		60.67 −2.26 (−3.07, −1.44)	<0.001	Gender, ethnicity, caregiver, marital status, psych service use, education session	−1.87 (−2.79, −0.96)	<0.001
BDI in 6 categories (per category) – low score to high score**		49.20 0.60 (−0.43, 1.63)	0.25	Age, employment, diabetes, API IS	−0.28 (−1.33, 0.77)	0.61
Anxiety State in 4 categories (per category) – low score to high score**		49.56 0.47 (−1.14, 2.08)	0.56	Age, employment, ethnicity, marital status	−0.35 (−1.92, 1.22)	0.66
Anxiety Trait in 4 categories (per category) – low score to high score**		48.55 0.82 (−0.75, 2.38)	0.31	Employment, ethnicity, marital status, CCI, API IS	−0.60 (−2.18, 0.98)	0.46
3MS in 5 categories (per category) – high score to low score**		51.64 −0.63 (−2.49, 1.22)	0.50	Age, employment, diabetes, IHD, TMT A	0.98 (−1.03, 2.99)	0.31
Options education session	No	51.29 (49.40, 53.18)	0.11	Age, employment, group	0.11 (−3.73, 3.96)	0.95
	Yes	48.13 (44.77, 51.48)				
Predialysis education experience	Very poor/ Not useful/ Inadequate	52.53 (46.56, 58.49)	0.18	Employment, caregiver, marital status, psych service use, education session, group	1.95 (−4.91, 8.80) 1.58 (−2.30, 5.45)	0.69
	Good	50.72 (48.03, 53.40)				
	Excellent	47.62 (44.79, 50.45)				
TMT A (per unit)**		54.21 −0.07 (−0.13, −0.01)	0.018	Age, marital status, psych service use, diabetes, IHD	−0.01 (−0.07, 0.05)	0.78
TMT B (per unit)**^∆^		54.08 −0.02 (−0.06, 0.01)	0.22	Age, ethnicity, marital status, psych service use, diabetes, IHD, TMT A	---	---
API IS	Per percentage increase	26.56 0.30 (0.15, 0.44)	<0.001	Age, group	0.19 (0.04, 0.33)	0.010
Group	Predialysis Hospital Self-care	45.88(43.32, 48.44) 51.96 (49.43, 54.48) 56.81 (53.20, 60.41)	<0.001	Age, caregiver, marital status, psych service use, diabetes, education session, TMT A, API IS	−7.09 (−12.09, −2.09) −1.76 (−6.82, 3.29)	0.006

*Estimated Marginal Mean

**The results for these continuous variables are presented as the intercept, the parameter estimate and the 95 % CI of the parameter estimate

∆ Analysis not reported as >25 % missing values in the dataset

**Table 3:** Multivariable Analysis (Age, education and group included a priori)

MULTIVARIABLE LINEAR REGRESSION ANALYSIS: DECISION-MAKING SUBSCALE			
Variable		Parameter estimate (95 % CI)	*p*-value
Education	High school	−1.23 (−4.83, 2.36)	0.50
	Post high school	~	
Group	Predialysis	−8.20 (−12.59, −3.81)	<0.001
	In-centre HD	−1.68 (−6.05, 2.68)	
	Home HD	~	
Gender	Male	−3.29 (−6.52, −0.07)	0.046
	Female	~	
Marital Status	Married or Partner	8.79 (2.92, 14.65)	0.015
	Single	5.43 (−1.35, 12.21)	
	Divorced/Separated	6.26 (−1.28, 13.80)	
	Widowed	~	
Age (per 10 years)		−3.27 (−4.54, −2.01)	<0.001
Ethnicity	White	10.62 (5.36, 15.89)	<0.001
	Non-white	~	
API (Information Seeking %)	Per percentage increase	0.15 (0.01, 0.30)	0.035
Between-centre variability		1.17 X 10^−19^ (1.05 X 10^−36^, 0.01)	-
Within-centre variability		253.25 (221.53, 289.52)	-
Intra Class Coefficient (ICC)***		4.61 X 10^−22^ (−)**	-
MULTIVARIABLE LINEAR REGRESSION ANALYSIS: INFORMATION-SEEKING SUBSCALE			
Variable		Parameter estimate (95 % CI)	p-value
Education	High school	−2.06 (−4.41, 0.28)	0.085
	Post high school	~	
Cohort	Predialysis	−4.16 (−6.99, −1.33)	<0.001
	In-centre HD	−0.20 (−3.05, 2.64)	
	Home HD	~	
Marital status	Married or partner	1.84 (−1.96, 5.65)	0.002
	Single	−2.56 (−6.96, 1.83)	
	Divorced or sep.	4.14 (−0.75, 9.04)	
	Widowed	~	
Age (per 10 years)		−0.79 (−1.60, 0.03)	0.058
BDI in 6 categories (per category increase)*		0.86 (0.24, 1.49)	0.007
Between-centre variability		1.14 (0.08, 16.36)	-
Within-centre variability		107.87 (94.40, 123.27)	-
Intra Class Coefficient (ICC)***		0.01 (7.28 X 10^−4^, 0.13)	-

*BDI categories: 0–10, 11–15, 16–20, 21–25, 26–30, ≥31

**Standard error estimate of ICC very close to 0 so no confidence interval provided

***ICC is a measure of the correlation of observations in the same cluster. ICC close to 1 indicates the people in the cluster are very similar, whereas ICC close to 0 indicates the between cluster variability is small compared to the within cluster variability

### ‘Autonomists’ vs ‘Delegators’

**Fig 4:**
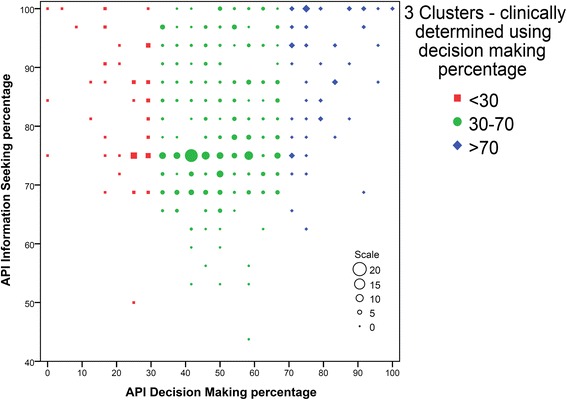
Distribution of patient clusters determined by high and low decision-making scores

Decision-making subscale scores were subdivided into tertiles to identify variables associated with high (DM > 70: empirically designated as autonomists) and low DM (≤30: empirically designated as delegators) scores (Fig. 4). This shows association of higher decision-making scores with lower age, lower comorbidity index scoring, higher executive brain function, belonging in the self-caring cohort and being unemployed (although lack of employment may have been a conscious decision of the study participants) (Table 4). Some of these variables separate the two patient clusters (e.g. CCI, higher cognitive scores etc.), but these have not featured in the final multivariable model involving the total patient cohort, possibly due to differences being most extreme at very high and very low scores.

**Table 4:** Cluster associations with demographic, clinical and psychosocial variables

Variable	1(Delegators) (*n* = 57)	3(Autonomists) (*n* = 66)	*p*-value
Group			
Predialysis	30 (52.6 %)	15 (22.7 %)	<0.001^b^
Hospital	19 (33.3 %)	27 (40.9 %)	
Home	8 (14.0 %)	24 (36.4 %)	
Age- Median (IQR)	67.0 (56.0-71.5)	52.0 (42.8-63.0)	<0.001^a^
Employment			
Retired	39 (68.4 %)	22 (33.3 %)	<0.001^b^
Unemployed	6 (10.5 %)	24 (36.4 %)	
Self-employed	4 (7.0 %)	4 (6.1 %)	
Salaried	8 (14.0 %)	16 (24.2 %)	
Marital Status			
Married or partner	34 (59.6 %)	43 (65.2 %)	0.051^b^
Single	9 (15.8 %)	15 (22.7 %)	
Divorced or separated	4 (7.0 %)	6 (9.1 %)	
Widowed	10 (17.5 %)	2 (3.0 %)	
CCI* Median (IQR)	*n* = 56 5.0 (4.0-7.0)	*n* = 62 3.0 (2.8-5.0)	<0.001^a^
TMT A Median (IQR)	*n* = 56 49.0 (38.0-61.5)	*n* = 64 37.5 (30.5-52.3)	0.008^a^
TMT B Median (IQR)	*n* = 43 105.0 (84.0-137.0)	*n* = 54 72.0 (59.0-122.5)	0.007^a^
IS score Median (IQR)	*n* = 57 84.38 (75.00-90.63)	*n* = 66 93.75 (81.25-100)	<0.001^a^

*CCI: Charlson Comorbidity Index

^a^Mann-Whitney *U* test p-value

^b^Fisher’s exact test p-value

Note: A sensitivity analyses was carried out with cut-off scores for the API-DM subscale for patient subgroups at 25/75 and at 35/65.The significant variable outcomes across all these analyses are comparable (Additional file 3: supplementary material provided)

### Decision-making in a prospective observation of the subset of dialysis starters

Complete API data on dialysis starters was available from 30 predialysis patients who commenced dialysis during the follow-up period up to 1 year from study entry. The data was completed at least 3 months after commencement of dialysis. The mean (SD) decision-making percentage score after commencement of dialysis was 37.79 (16.45), which was not significantly different from the predialysis mean (SD) of 40.52(11.73).

## Discussion

Clinical outcomes associated with RRT modalities are different. The challenge with interpreting reported outcomes on modality superiority are the sociodemographic, physiological and psychological differences which exist between patients in different treatment groups, and the change over time in some of these factors. The systematic exclusion, through lack of information of patient’s values, preferences and engagement, leading to the choice (or the lack of one) of modality may also have a bearing on the desired outcomes. In our study of patient preferences for autonomous decision-making in ESRD management, we have a large, representative sample population, including predialysis patients in the process of modality decision-making and patients established on ‘in-centre’ and ‘self-care’(predominantly home-based) haemodialysis, from across five tertiary centres. To our knowledge, this is the first study which has examined the issue of patient preferences for information-seeking and decision-making in an ESRD population preparing to receive dialysis and that in receipt of haemodialysis therapy, simultaneously. We have also examined longitudinally, if DM preference in predialysis patients changes over time in a subset of patients, after commencement of dialysis therapy. The variables considered for analyses have been categorised in a manner meaningful for clinical interpretation. These apply especially to neuropsychometric tests and depression and anxiety screening tools where use of cut-off points may result in loss of information to be ascertained from scores further removed from the cut off mark. The coefficient of internal consistency of the two subscales of API is high, ensuring reliability of the test findings in our study population.

In a separate study by Flynn et al.[24], cluster analysis was used to understand the typology of patient preferences in a large group of older adults. The vast majority of them wanted information exchange, but differed in preferences for discussing and selecting treatment choices based on deliberation and decisional control. This study highlighted the need for strategies to improve information exchange and distinguish preferences for discussing and selecting treatment options. Our study has taken the understanding of the subject to individuals who are on a declining health course, those in receipt of different treatment types, respondents in varied sociodemographic groups and considered varied cognitive and psycho-affective factors which may also influence the actual response outcomes.

### Demographic variables and patient preference for decision-making in ESRD

Age is an important factor in decision-making preference, with younger age group preferring a more active role in decision-making. Despite the fact that older patients wanted less participation in treatment decision-making, they nonetheless wanted a similar degree of information (high overall median scores on information-seeking subscale), demonstrating that wanting information and making decisions are separate constructs[25]. Even amongst the study cohort of high information-seekers overall, age still emerged as a significant factor for both information-seeking and decision-making, with high scores in favour of lower age. Higher confidence, greater overall perceived knowledge, greater overall retained information/knowledge and access to modern educational resources (internet), consulted by the younger population may play a role, in this regard. Besides, the type of information required by the older age group and the manner in which information is provided to older patients may have to be tailored to individual preferences, such that, their engagement with the decision-making process is well facilitated and meaningful. Higher education, like age, was forced into our model for multivariate regression analysis and did emerge as a significant variable for information-seeking, but not for decision-making. This highlights the role of the individual’s coping style and the complexity of ESRD decision-making process, making collaborative decision-making, a preferred route even amongst those who are well educated and actively seek involvement through information. Even in the cluster examination of ‘autonomists’ vs ‘delegators’, education, was not associated with either category. This is contrary to what has been noted in some other conditions where API was used to study decision-making preference [26–28] and also in other qualitative research in the area of medical decision-making[29]. Several studies have identified that gender is associated with DM preference, all finding that women are more likely than men to prefer a more active role[30]. This finding has been replicated in our ESRD study population too, although another observational study in ESRD patients, did not find significant gender differences, although a smaller study population may explain this [31]. The role of gender and the biology of decision-making remains an interesting area of research, but, the influence of gender on interpersonal relationship between the physician and the patient may influence participatory decision-making styles [32]. Employment is not a significant predictor on multivariate analysis, but remains a significant association in the cluster of low decision-making scorers. This is likely to be due to the fact that the employment variable is very closely linked to the ‘cohort’ variable, which was a significant predictor on the regression analysis. The cluster association showed that ‘retired’ individuals were more likely to assume a passive role in decision-making. Significantly higher proportion (37 %) of unemployed participants were found in the ‘autonomists’ group, although the decision to stay unemployed may have been taken consciously by this group. Marital status also seems to influence decision-making preference, with married individuals more likely than unmarried, divorced or widowed participants to play an active role in decision-making. Ethnicity is associated with DM in our study, with white patients more likely to prefer to be involved in decision-making. Patient’s role expectations, perceived role in the family context and emphasis on individuality may be a culturally determined phenomenon, influencing the passive role adopted by participants in the ethnic minority group [33]. The approach to imparting information and ascertaining patient’s values and preferences should be culturally sensitive and account for the cultural diversity of different regions.

### Cognitive function and decision-making preference in ESRD

We examined the association of scores from Trail making tests A and B and 3MS, a test of global cognition, with decision-making preference scores. The lowest tertile of decision-making scores was associated with poorer scores on the tests of executive brain function. Although this was not significant in the multivariate regression analysis, TMT A scores were significant on single variable analysis. This is likely due to a significant proportion of missing data on TMTs, largely due to patients’ inability to complete tests or unwillingness to undertake the tests due to perceived complexity. There is evidence from literature linking age, cognition and other individual resources with health literacy in advanced age[12]. Results show that executive function and episodic memory explained literacy decline with age considerably. Executive function also had an indirect effect via risk aversion. The finding that impaired health literacy in old age is in part a function of cognitive decline even amongst persons without dementia, has clear implications for policy and intervention. Thus, it is high priority to reduce cognitive demands, particularly complex reasoning abilities and memory, inherent in the health literature materials and decision-making aids used by patients with even milder degrees of cognitive impairment. Learning styles specific information and reinforcement of consistent messages will ensure correct understanding. Impact of depression on DM was not significant in our study, but higher BDI scores were significantly associated with higher information-seeking. The ability to appreciate, understand the significance, express choice or engage in a logical process of analysing the information ascertained are known to be impaired in depressed patients in other studies [34].

### Illness burden and decision-making preference in ESRD

None of the comorbidities emerged significant predictors of decision-making in the multivariate analysis. However, the Charlson comorbidity index score, a prognostic tool, was significant in the univariate analysis, and so were, diabetes, heart failure and ischaemic heart disease. In the cluster examination, the lowest tertile of decision-making scores was associated significantly with high comorbidity burden. The impact of illness on decision-making is difficult to dissociate from the role of medical care received for the illness on decision-making. Results from studies in published literature suggest that patient’s preference may change in time as their experience of illness evolves [35] and that, experiences of interactions with healthcare providers may also affect patient’s desire to involve themselves in current or future decision-making[26]. It is apparent from our study that in the small subset of predialysis patients, who were re-assessed at least three months after commencement of dialysis therapy, no significant change in their decision-making preferences, was observed. It is also apparent that the more complex or urgent the clinical condition, the more likely ESRD patients would consider adopting a more passive role.

### Information preference and study group influence

Our study demonstrates that there is great appetite for information across all study groups. Even amongst the high information-seekers, API information-seeking score greater than 75, is associated significantly with greater preference for decision-making. The scores are significantly higher in the self-caring cohort and this may well be associated with an active coping style, the same group demonstrating higher preference for involvement in decision-making. The predialysis group was more likely to want shared involvement in the decision-making process compared to other groups in the multivariate analysis and cluster association of low decision-makers. The lack of concrete, personal experience of the treatment process may be the reason for their concern. Therefore, revisiting treatment options after commencement of dialysis may influence the choice of long-term dialysis treatment considerations including location and self vs shared vs institutional care.

### Links with medical humanities and social sciences

The study findings are well in line with predictions drawn from established theories in social psychology. For instance, decision fatigue [36]is a psychological state, where the ability to process complex information and to make autonomous decisions is depleted due to e.g. emotional upheaval, resulting in impulsiveness, evasive behaviour or helplessness. The effects of illness burden on decision-making preference in ESRD can be seen correlating with both decision fatigue and emotional adjustment[37, 38]: a patient who has been only recently diagnosed with a severe illness is emotionally and cognitively handicapped due to mental fatigue, in comparison to a patient who has had time to process the emotional upheaval and adjust. Recent developments in the psychology of decision-making have revealed several factors influencing and distorting the ability to make autonomous, well-informed decisions, of which decision fatigue is only one. To facilitate patient autonomy in various stages of emotional adjustment and levels of fatigue, procedures including psychological support and appropriate information design become necessary to ensure the fulfillment of patient autonomy.

### Practical implications of the knowledge of patient’s decision-making preference

It is known from published literature that patients who are educated about all of their treatment options are significantly more likely to choose a home-based treatment option [39–41]. Information empowers patients to choose their RRT modality. The manner in which this information is presented therefore would influence the patients’ choice of therapy. Many decisions of this complexity may well result in a shift in decision-making equipoise, making patient-led autonomous decisions, a function, limited by three key factors-patient characteristics, time constraints and clinical urgency. It is apparent from our study that subsets of well-informed patients are still keen on involving the healthcare team in their decision-making process. There are patient characteristics which influence their wish to be involved in decision-making. These become apparent as the clinical encounter progresses over time. The reasons behind delegating the choice to another person need to be explored at a clinical and psychosociocultural level through collaborative decision-making, engaging patients, their family, and several members of the multi-disciplinary care team. This process typically operates in considerable time constraints, making a truly autonomous decision or shared decision-making by patient choice, an option for a limited few. The third point on clinical urgency is a situation where patients naturally lean towards their physician in making the right choices for them. Presenting all dialysis options as equal with the healthcare team remaining modality neutral (and therefore presumed unbiased), without clarifying the impact of each choice on the course of their illness, associated morbidity, mortality and quality-of-life, renders modality education practice unchanged and unresponsive to published scientific literature. Furthermore, patient’s decision-making preferences ought to be juxtaposed to the systematic assessment of patients’ affect and cognitive abilities and actual as against perceived knowledge. These remain integral to understanding the level and duration of healthcare provider engagement required to facilitate literacy and the decision process.

### Study limitations

There are limitations to our study. Assessing healthcare provider’s decision-making preferences would be important as decision-making happens during this bidirectional exchange of information. Assessment of actual knowledge as a predictor of decision-making preference would be useful. Although a number of clinical, psychological and socio-demographic variables have been considered, autonomy preference in a medical context is likely to be influenced by immeasurable factors and therefore our findings do not necessarily present an exhaustive list of predictors of autonomy preference in ESRD or explain the variance in autonomy preference. It is also not possible to ascertain from our study if preferred participation differs from actual participation levels when removed from hypothetical scenarios.

## Conclusions

The study explored decision-making preferences and its influencing factors in ESRD patients overall and according to their position with respect to dialysis commencement. ESRD patients prefer to receive information, but this does not always translate into active involvement in decision-making. This may not be acceptable or appropriate for everyone and the patient may choose to determine the extent to which they seek involvement. By identifying factors which might affect patient preference for involvement, health professionals may move away from a normative, ‘one size fits all’ approach, be more sensitive to individual patient’s preferences and provide better patient-centred; individual-appropriate care.

## Additional files

Additional file 1: **SUPPLEMENTARY MATERIAL Missing data analysis.** (DOCX 15 kb)

Additional file 2: **SUPPLEMENTARY MATERIAL-1. TABLE : Item statistics for both subscales.** (DOCX 16 kb)

Additional file 3: **Sensitivity Analysis: Cluster Analysis with different API-DM Score cut-offs.** (DOCX 29 kb)

## Abbreviations

3MS: Modified Mini Mental State
API: Autonomy Preference Index
BASIC-HHD: Barriers to Successful Implementation of Care in Home Haemodialysis
CCI: Charlson Comorbidity Index
CKD: Chronic Kidney Disease
DM: Decision-Making
ESRD: End Stage Renal Disease
HD: Haemodialysis
IS: Information-Seeking
NRES: National Research Ethics Service
PD: Peritoneal Dialysis
TMTA/B: Trail Making Tests A/B
BDI: Beck Depression Inventory
STAI: State and Trait Anxiety Inventory
