# Supplementary material for: Healthcare decision-making in end stage renal disease-patient preferences and clinical correlates
Source: BMC Nephrol. 2015 Nov 14;16:189. doi: 10.1186/s12882-015-0180-8 (PMC4647276; doi:10.1186/s12882-015-0180-8)
Supplement: Supplementary file 1 — SUPPLEMENTARY MATERIAL Missing data analysis. (DOCX 15 kb) [file 12882_2015_180_MOESM1_ESM.docx]

SUPPLEMENTARY MATERIAL Missing data analysis

| Variable | Not missing (n=458) | Missing (n=77) | p-value |
| --- | --- | --- | --- |
| Cohort  Predialysis  In-centre HD  Home HD | 182 (39.7%)  186 (40.6%)  90 (19.7%) | 40 (51.9%)  27 (35.1%)  10 (13.0%) | 0.11^2^ |
| Age  Mean (std. dev.) | 57.04 (13.90) | 54.01 (12.97) | 0.075^1^ |
| Gender – male | 296 (64.6%) | 49 (63.6%) | 0.87^2^ |
| Education – post-high school | 111/442 (25.1%) | 22/74 (29.7%) | 0.40^2^ |
| Employment  Retired  Unemployed  Self-employed  Salaried | 213/455 (46.8%)  115/455 (25.3%)  35/455 (7.7%)  92/455 (20.2%) | 25 (32.5%)  27 (35.1%)  6 (7.8%)  19 (24.7%) | 0.11^2^ |
| Ethnicity – non-white | 46/457 (10.1%) | 15 (19.5%) | 0.016^2^ |
| BMI (kg/m^2^)  Median (IQR) | n=455  27.18 (23.85-31.97) | 27.73 (24.33-32.64) | 0.51^3^ |
| Smoking status  Never smoked  Ex-smoker  Current | 257/453 (56.7%)  133/453 (29.4%)  63/453 (13.9%) | 44/75 (58.7%)  18/75 (24.0%)  13/75 (17.3%) | 0.55^3^ |
| Marital status  Married or partner  Single  Divorced/separated  Widowed | 280 (61.1%)  103 (22.5%)  40 (8.7%)  35 (7.6%) | 43 (55.8%)  21 (27.3%)  8 (10.4%)  5 (6.5%) | 0.74^2^ |
| Diabetes – yes | 123/455 (27.0%) | 28/76 (36.8%) | 0.079^2^ |
| CCI  Median (IQR) | n=443  4 (3-6) | n=75  4 (3-6) | 0.55^3^ |
| BDI  Median (IQR) | 10.0 (5.0-19.0) | n=22  12.0 (0.0-18.3) | 0.70^3^ |
| TMT A  Median (IQR) | n=427  45.0 (33.0-60.0) | n=68  39.5 (29.0-57.0) | 0.11^3^ |
| TMT B  Median (IQR) | n=338  90.0 (69.0-122.3) | n=55  87.0 (67.0-120.0) | 0.65^3^ |

^1^t-test ^2^Pearson chi-squared test ^3^Mann-Whitney U test

The only statistically significant difference between those who were missing both the API decision making and API information seeking scores and those who were not missing both is in ethnicity. Non-white patients were more likely not to complete both API scores than white patients. Ethnicity was associated with decision making in the final multivariable analysis for the decision making variable. Therefore, there is a chance that the point estimate may change slightly, depending on whether the non-white patients who responded had different scores to those who did not respond. However, with the relatively small amount of missing data and only 15 non-white patients not having either score, any change would be small. There was no relationship in the single variable analysis between ethnicity and information seeking so unless the missing non-white patients differed greatly to the non-white patients who responded, it is likely the lack of association would remain.
