# Supplementary material for: Healthcare decision-making in end stage renal disease-patient preferences and clinical correlates
Source: BMC Nephrol. 2015 Nov 14;16:189. doi: 10.1186/s12882-015-0180-8 (PMC4647276; doi:10.1186/s12882-015-0180-8)
Supplement: Supplementary file 2 — SUPPLEMENTARY MATERIAL-1. TABLE : Item statistics for both subscales. (DOCX 16 kb) [file 12882_2015_180_MOESM2_ESM.docx]

SUPPLEMENTARY MATERIAL-1

**TABLE : Item statistics for both subscales**

| **INFORMATION-SEEKING SUBSCALE (N=452)**  **(CRONBACH’S ALPHA 0.774)** | | | |
| --- | --- | --- | --- |
|  | | **Mean** | **Std. Deviation** |
| I7 | | 3.04 | .831 |
| I8 | | 3.21 | .762 |
| I9 | | 3.41 | .602 |
| I10 | | 3.34 | .545 |
| I11 | | 2.86 | 1.001 |
| I12 | | 3.34 | .638 |
| I13 | | 3.27 | .692 |
| I14 | | 3.46 | .529 |
| **DECISION-MAKING SUBSCALE (N=451)**  **(CRONBACH’S ALPHA 0.714)** | | | |
|  | **Mean** | | **Std. Deviation** |
| D1 | 1.98 | | 1.264 |
| D2 | 2.13 | | 1.170 |
| D3 | 2.32 | | 1.189 |
| D4 | 2.60 | | 1.001 |
| D5 | 1.27 | | .942 |
| D6 | 1.82 | | 1.074 |
