# Supplementary material for: Healthcare decision-making in end stage renal disease-patient preferences and clinical correlates
Source: BMC Nephrol. 2015 Nov 14;16:189. doi: 10.1186/s12882-015-0180-8 (PMC4647276; doi:10.1186/s12882-015-0180-8)
Supplement: Supplementary file 3 — Sensitivity Analysis: Cluster Analysis with different API-DM Score cut-offs. (DOCX 29 kb) [file 12882_2015_180_MOESM3_ESM.docx]

Patient subgroups

| **Cluster** | Cluster size  (N=451) | Decision Making percentage  Mean (standard deviation)  Median (IQR and range) | Information Seeking percentage  Mean (standard deviation)  Median (IQR and range) |
| --- | --- | --- | --- |
| **1**  **DM≤25** | 35 (7.8%) | 18.21 (7.77)  20.83 (16.67-25.00, 0-25) | 82.86 (11.65)  84.38 (75.00-90.63, 50.00-100) |
| **2**  **25<DM<75** | 368 (81.6%) | 49.41 (11.89)  50.00 (41.67-58.33, 29.17-70.83) | n=362  79.74 (10.67)  75.00 (71.88-87.50, 43.75-100) |
| **3**  **DM≥75** | 48 (10.6%) | 82.64 (7.75)  79.17 (75.00-87.50, 75.00-100) | 90.76 (9.78)  93.75 (84.38-100, 62.50-100) |

Patient subgroup characteristics

| Variable | 1 (n=35) | 3 (n=48) | p-value |
| --- | --- | --- | --- |
| Cohort  Predialysis  Hospital  Home | 19 (54.3%)  14 (40.0%)  2 (5.7%) | 9 (18.8%)  23 (47.9%)  16 (33.3%) | 0.001^2^  Predialysis  1>3 <0.05^3^  Home  1<3 <0.05^3^ |
| Age  Median (IQR) | 67.0 (56.0-72.0) | 52.0 (40.0-63.0) | <0.001^1^ |
| Employment  Retired  Unemployed  Self-employed  Salaried | 26 (74.3%)  3 (8.6%)  1 (2.9%)  5 (14.3%) | 16 (33.3%)  19 (39.6%)  2 (4.2%)  11 (22.9%) | 0.001^4^  Retired  1>3 <0.05^3^  Unemployed  1<3 <0.05^3^ |
| Marital Status  Married or partner  Single  Divorced or sep  Widowed | 20 (57.1%)  6 (17.1%)  2 (5.7%)  7 (20.0%) | 30 (62.5%)  12 (25.0%)  4 (8.3%)  2 (4.2%) | 0.16^4^ |
| CCI  Median (IQR) | n=34  5.0 (4.0-7.0) | n=45  4.0 (2.5-5.0) | 0.001^1^ |
| TMT A  Median (IQR) | n=34  51.5 (35.3-64.8) | n=47  38.0 (32.0-50.0) | 0.038^1^ |
| TMT B  Median (IQR) | n=25  120.0 (82.0-148.5) | n=41  74.0 (59.5-142.0) | 0.11^1^ |
| API IS (percentage)  Median (IQR) | 84.38 (75.00-90.63) | 93.75 (84.38-100) | 0.001^1^ |

^1^Mann-Whitney U test ^2^Pearson chi-squared test

^3^z-test comparing category proportions between groups with Bonferroni adjustment for multiple testing

^4^Fisher’s exact test

Patient subgroups

| **Cluster** | Cluster size  (N=451) | Decision Making percentage  Mean (standard deviation)  Median (IQR and range) | Information Seeking percentage  Mean (standard deviation)  Median (IQR and range) |
| --- | --- | --- | --- |
| **1**  **DM≤35** | 92 (20.4%) | 26.59 (8.28)  29.17 (25.00-33.33, 0-33.33) | n=91  82.04 (10.44)  81.25 (75.00-90.63, 50.00-100) |
| **2**  **35<DM<65** | 258 (57.2%) | 49.48 (7.67)  50.00 (41.67-54.17, 37.50-62.50) | n=254  78.70 (10.83)  75.00 (71.88-87.50, 43.75-100) |
| **3**  **DM≥65** | 101 (22.4%) | 75.00 (9.15)  70.83 (66.67-79.17, 66.67-100) | n=100  86.66 (10.72)  87.50 (78.13-96.88, 62.50-100) |

Patient subgroup characteristics

| Variable | 1 (n=92) | 3 (n=101) | p-value |
| --- | --- | --- | --- |
| Cohort  Predialysis  Hospital  Home | 48 (52.2%)  32 (34.8%)  12 (13.0%) | 25 (24.8%)  45 (44.6%)  31 (30.7%) | <0.001^2^  Predialysis  1>3 <0.05^3^  Home  1<3 <0.05^3^ |
| Age  Median (IQR) | 66.0 (56.0-71.8) | 52.0 (44.0-59.5) | <0.001^1^ |
| Employment  Retired  Unemployed  Self-employed  Salaried | n=91  61 (67.0%)  9 (9.9%)  7 (7.7%)  14 (15.4%) | 32 (31.7%)  38 (37.6%)  10 (9.9%)  21 (20.8%) | <0.001^2^  Retired  1>3 <0.05^3^  Unemployed  1<3 <0.05^3^ |
| Marital Status  Married or partner  Single  Divorced or sep  Widowed | 53 (57.6%)  17 (18.5%)  8 (8.7%)  14 (15.2%) | 65 (64.4%)  24 (23.8%)  9 (8.9%)  3 (3.0%) | 0.027^2^  Widowed  1>3 <0.05^3^ |
| CCI  Median (IQR) | n=91  5.0 (4.0-7.0) | n=95  4.0 (3.0-5.0) | <0.001^1^ |
| TMT A  Median (IQR) | n=86  49.5 (37.5-63.3) | n=95  39.0 (32.0-53.0) | 0.005^1^ |
| TMT B  Median (IQR) | n=66  103.0 (73.4-134.8) | n=80  78.5 (61.3-117.0) | 0.018^1^ |
| API IS (percentage)  Median (IQR) | n=91  81.25 (75.00-90.63) | n=100  87.50 (78.13-96.88) | 0.003^1^ |

^1^Mann-Whitney U test ^2^Pearson chi-squared test ^3^z-test comparing category proportions between groups with Bonferroni adjustment for multiple testing
